# 

**Published:** 1818-01

**Authors:** 


### Medico-Chirurgical

### JOURNAL AND REVIEW.

CONTAINING,

I. Select Original Communications.

II. Comprehensive Analytical Review of all important Medical Publications; presenting a fair Epitome of the Medical Literature of the Day.

III. Select Translations from Foreign Medical Journals.

IV. Medical Miscellanies, Foreign and Domestic; Anonymous Communications, &c. &c.

CONDUCTED BY

WILLIAM SHEARMAN, M. D.

Licentiate of the Royal College of Physicians; Physician to the London Dispensary; and to the Universal Dispensary for Children;

JAMES JOHNSON, Esq.

Consulting Surgeon to His Royal Highness the Duke of Clarence; Author of "The Influence of Tropical Climates on European Constitutions;" and of "The Influence of the Atmosphere on the Health and Functions of the Human Frame," &c.;

AND

SHIRLEY PALMER, M. D.

Of the University of Glasgow.

OMNIA VINCIT LABOR SI LABOR IPSE VOLUPTAS.

VOL. V.

From January to June, 1818.

103676

447

S.G.O. LIBRARY 63388

*LONDON.*

PRINTED FOR THE PROPRIETORS,

BY WILLIAM THORNE, RED LION COURT, FLEET STREET.

To whom Communications, addressed to the Editors, are requested to be sent.

Sold by Baldwin, Cradock, and Joy, Pater-Noster Row; Highley and Son, and T. & G. Underwood, Fleet Street; J. Callow, Crown Court, Soho; Cox and Son, Borough; Anderson and Chase, West Smithfield; W. Blackwood, Edinburgh; J. Cumming, Dublin; and may be had also by applying to the Clerks of the Roads, at the General Post Office.

### CORRESPONDENCE.

Dr. Moulson's Paper on Typhus, Medical Report of H. M. S. Venerable, &c. have been received ; as has also Dr. Crichton's Account of some Experiments with the vapour of Pitch.

Mr. Cunningham's very interesting Communication, relative to a new method of treating extensive abscesses, has come to hand, and will be published as soon as an overflow of previously received matter can be disposed of.

"A Country Practitioner's" strictures on the contemptible production entitled A Picture of the present State of the Royal College of Physicians of London, are very just, although we have not room to insert them. In answer to his inquiry, where he can find the *hepatic theory of disease*, we must refer him to the writings of Dr. Curry, to whom the origin of that theory is attributed, in the book just mentioned.

A copious Review of Dr. Bancroft's Sequel to his Essay on Fever, will appear in our next.

In answer to our numerous Correspondents, who complain of our not noticing the case of the lamented Princess Charlotte of Wales, we must reply, that as no authentic statement of the facts has yet been given by those who are competent to do it, the medical gentlemen in attendance, we are unwilling to fill our pages with mere *newspaper gossip*. Whenever such authentic statement shall appear, and we are among those who think the public have a right to expect it, we shall not fail to present it to our readers, together with such remarks and observations as an impartial review of the whole case will enable us to form.

The Editors beg leave to inform Dr. Moulson, that the substitution of two other Journals successively, for the Medico-Chirurgical Journal, arose entirely from the mistake of his own bookseller, in not copying the title of the work accurately in the order he gave, as they had an opportunity of ascertaining by an inspection of the order in the counting-house of Longman and Co. It is earnestly requested that Subscribers and Correspondents will be precise in their orders for the Medico-Chirurgical Journal, as mistakes frequently arise from inaccurate titles given by country booksellers—Should any difficulty be experienced in obtaining the work, the Editors will, upon receiving information, make enquiries into the causes: and, to the utmost of their power, obviate the recurrence of disappointment.

---

### *Diseases and Casualties in London in the Year* 1817.

| | |
|---|---|
| Abortive and Stillborn | 700 |
| Abscess | 98 |
| Aged | 1875 |
| Ague | 2 |
| Apoplexy & Suddenly | 462 |
| Aſthma | 743 |
| Bedridden | 5 |
| Bile | 6 |
| Bleeding | 45 |
| Burſten and Rupture | 43 |
| Cancer | 99 |
| Chicken Pox | 1 |
| Childbed | 252 |
| Colds | 14 |
| Colic, Gripes, &c. | 7 |
| Conſumption | 4200 |
| Convulſions | 3242 |
| Cough, and Hooping Cough | 645 |
| Cow Pox | 1 |
| Cramp | 1 |
| Croup | 109 |
| Diabetes | 3 |
| Dropſy | 718 |
| Epilepſy | 5 |
| Evil | 6 |
| Fevers of all kinds | 1299 |
| Fiſtula | 3 |
| Flux | 9 |
| French Pox | 86 |
| Gout | 54 |
| Gravel, Stone, and Strangury | 24 |
| Grief | 4 |
| Headmoldſhot, Horſeſhoe head, and water in the head | 419 |
| Impoſthume | 2 |
| Inflammation | 1002 |
| Jaundice | 75 |
| Jaw Locked | 2 |
| Livergrown | 76 |
| Lumbago | 1 |
| Lunatick | 244 |
| Meaſles | 725 |
| Miſcarriage | 6 |
| Mortification | 304 |
| Palpitation of the Heart | 4 |
| Palſy | 162 |
| Pleuriſy | 22 |
| Purples | 1 |
| Quinſy | 2 |
| Raſh | 2 |
| Rheumatiſm | 11 |
| Scrophula | 5 |
| Scurvy | 6 |
| Small Pox | 1051 |
| Sore Throat | 5 |
| Sores and Ulcers | 11 |
| Spaſm | 25 |
| St. Anthony's Fire | 7 |
| Stoppage in the Stom. | 24 |
| St. Vitus's Dance | 1 |
| Stricture | 1 |
| Swelling | 1 |
| Teeth | 449 |
| Thruſh | 111 |
| Tumor | 3 |
| Water in the Cheſt | 68 |
| Worms | 12 |

| | |
|---|---|
| Broken Limbs | 6 |
| Bruised | 2 |
| Burnt | 41 |
| Choaked | 2 |
| Drowned | 119 |
| Exceſſive Drinking | 12 |
| Executed | 26 |
| Found Dead | 29 |
| Fractured | 4 |
| Frighted | 9 |
| Killed by Falls and ſeveral other Accidents | 65 |
| Killed themſelves | 34 |
| Murdered | 3 |
| Poiſoned | 6 |
| Scalded | 4 |
| Shot | 1 |
| Starved | 8 |
| Strangled | 1 |
| Suffocated | 11 |
| Total | 383 |

| | | | |
|---|---|---|---|
| Chriſtened | Males | 12624 | In all 24129 |
| | Females | 11505 | |
| Buried | Males | 10033 | In all 19968 |
| | Females | 9935 | |

Whereof have died,

| | | | |
|---|---|---|---|
| Under Two Years of Age | 5698 | Sixty and Seventy | 1614 |
| Between Two and Five | 2019 | Seventy and Eighty | 1224 |
| Five and Ten | 929 | Eighty and Ninety | 683 |
| Ten and Twenty | 706 | Ninety and a Hundred | 156 |
| Twenty and Thirty | 1364 | A Hundred | 7 |
| Thirty and Forty | 1795 | A Hundred and One | 0 |
| Forty and Fifty | 1983 | A Hundred and Three | 0 |
| Fifty and Sixty | 1788 | A Hundred and Five | 2 |

Decreaſed in the Burials this Year 348.

